# Effect of Diamond Polishing and Thermal Treatment on Carbon Paramagnetic Centers’ Nature and Structure

**DOI:** 10.3390/ma14247719

**Published:** 2021-12-14

**Authors:** Ira Litvak, Avner Cahana, Yaakov Anker, Sharon Ruthstein, Haim Cohen

**Affiliations:** 1 Department of Chemical Sciences, Faculty of Natural Sciences, Ariel University, Ariel 4077625, Israel; hcohen@ariel.ac.il; 2 The Israeli Diamond Bourse, Dianer Diamonds Company Ltd., Ramat Gan 5252005, Israel; dianerdiamonds@gmail.com; 3 The Eastern R&D Center, Ariel University, Ariel 4077625, Israel; kobia@ariel.ac.il; 4 Department of Chemical Engineering, Ariel University, Ariel 4077625, Israel; 5 Department of Chemistry and Institute of Nanotechnology & Advanced Materials, Faculty of Exact Sciences, Bar Ilan University, Ramat Gan 5290002, Israel; Sharon.ruthstein@biu.ac.il; 6 Department of Chemistry, Faculty Natural Sciences, Ben Gurion University of the Negev, Beer Sheva 8410501, Israel

**Keywords:** diamond treatments, EPR spectroscopy, paramagnetic centers

## Abstract

Diamonds contain carbon paramagnetic centers (stable carbon radicals) in small concentrations (at the level of ~1 × 10^12^ spins/mg) that can help in elucidating the structure of the nitrogen atoms’ contaminants in the diamond crystal. All diamonds that undergo polishing are exposed to high temperatures, owing to the friction force during the polishing process, which may affect the carbon-centered radicals’ concentration and structure. The temperature is increased appreciably; consequently, the black body radiation in the visible range turns orange. During polishing, diamonds emit an orange light (at a wavelength of about 600 nm) that is typical of a black body temperature of 900 °C or higher. Other processes in which color-enhanced diamonds are exposed to high temperatures are thermal treatments or the high-pressure, high-temperature (HPHT) process in which the brown color (resulting from plastic deformation) is bleached. The aim of the study was to examine how thermal treatment and polishing influence the paramagnetic centers in the diamond. For this purpose, four rough diamonds were studied: two underwent a polishing process, and the other two were thermally treated at 650 °C and 1000 °C. The diamonds were analyzed pre- and post-treatment by EPR (Electron Paramagnetic resonance), FTIR (Fourier transform infrared, fluorescence, and their visual appearance. The results indicate that the polishing process results in much more than just thermal heating the paramagnetic centers.

## 1. Introduction

Paramagnetic centers (stable radicals) in diamonds are produced upon exposure to high energetic interactions (e.g., high temperature, radiation, electrical discharges, or via chemical reactions). Diamonds can be exposed to these conditions via three main processes: (i) during volcanic eruptions, (ii) in controlled explosions during mining, and (iii) through diamond treatments—thermal treatments or HPHT, including polishing processes [1,2].

Diamonds contain mainly carbon-centered paramagnetic species [3]. The paramagnetic centers in diamonds, which are distributed in the crystal, occur both in rough and in polished diamonds. The most abundant paramagnetic centers in diamonds (rough or polished) are carbon-based; this refers to unpaired electrons centered on carbon atoms. These centers can be determined and studied by EPR (electron paramagnetic resonance) spectroscopy through the g-value property, which is a constant of proportionality (for a carbon g-value of 2.0028–2.0030). When nitrogen atoms (the main contaminants in diamonds [4]) are in the chemical neighborhood of the carbon-centered paramagnetic species in the diamond, they may affect the properties and the spectrum of the carbon-centered radicals. The paramagnetic centers in a diamond can interact with the ^14^N isotope (nuclear spin I = 1) and with the ^13^C isotope (nuclear spin I = 1/2). Moreover, paramagnetic centers may also appear, owing to a vacancy (V) (where a carbon atom is missing from the crystal lattice) [5,6,7,8,9,10].

**Figure 1:**
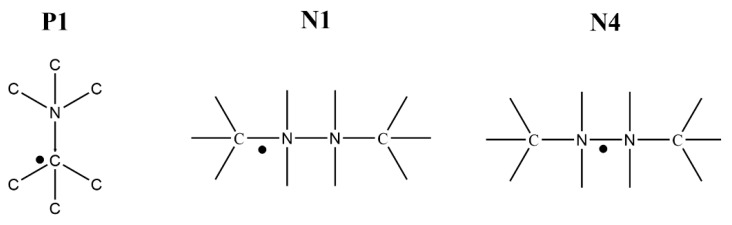
Structure of several paramagnetic centers: P1, N1, and N4.

In the past 60 years, these centers (carbon-centered and nitrogen-centered) have been characterized and cataloged according to the diamond type, the g-value, the hyperfine interactions, the treatments conducted on the diamond, and more. One of the most abundant and widely studied natural diamond paramagnetic centers is the P1 center (a single isomorphic N atom), close to the C-centered paramagnetic center (g = 2.0024 ± 0.0005) [11]. Sometimes the N1 paramagnetic center will appear together with the P1 center, which has the same g value, along with hyperfine constants that are about 10% larger than those of the nitrogen in the P1 center [12]. The N4 paramagnetic center, located on an N–N bond (g = 2.002 ± 0.001), is characterized by a lower g-value and hyperfine (A) constants [3,13,14]. The structures of several paramagnetic centers in N-contaminated diamonds are presented in Figure 1.

For diamond evaluation, four important properties were identified by the Gemological Institute of America in 1950: the four Cs—the carat, cut, clarity, and color of a diamond [15,16]. These properties determine the value and color grade for natural polished diamonds. Usually, the rating is from D color (which is the cleanest high value) up to N grade (which appears yellow-brown and has a much lower value). The color of the diamond usually results from the presence of elements in the diamond lattice such as nitrogen, boron, and others.

The most abundant contamination element in diamonds is nitrogen (occurring in ~95–97% abundance). The presence of nitrogen in diamonds indicates that the diamonds are classified as type I, whereas diamonds without any nitrogen contamination are classified as type II diamonds. In addition, different types of nitrogen centers occur in the crystal: the N type can be further classified into type Ia (aggregated nitrogen) and type Ib (isolated nitrogen). Type Ia can also be subclassified into IaA (two adjacent nitrogen atoms) and IaB (four nitrogen atoms surrounding a vacancy) [17]. Type II can also be classified into two groups: IIa—no presence of nitrogen—and IIb—the presence of boron atoms (very rare and are bluish gray). Diamonds can easily be classified into the correct type using FTIR) spectroscopy [18,19]. The nitrogen content and the fancy diamond type affect the color distribution that occurs across the entire visible spectral range [20].

This present work focuses on two high-energy reactions that may create changes in the paramagnetic centers of rough diamonds: the polishing process and thermal treatments.

Polishing single-crystal diamonds for the jewelry industry has been practiced for more than 3000 years; however, its origins remain largely unknown, probably because of the secrecy surrounding the diamond industry. Currently, the typical diamond polishing process involves pressing the crystal to a rotating ferrous wheel surface covered with diamond grits [21]. Furthermore, polishing is used to produce diamond-containing devices such as specialty semiconductors, optical windows, and heat diffusers [22,23].

One of the characteristics of diamond polishing is the anisotropy of the wear encountered. In the polishing trade, workers refer to the polishing directions in which wear proceeds fastest as ‘soft’, whereas directions in which little or no wear is induced are referred to as ‘hard’ [24]. Diamond anisotropy is a function of the contact pressure and the geometry of the sliding interface; it affects the friction coefficient. High friction coefficients were found in ‘soft’ sliding directions; conversely, low friction coefficients were observed in ‘hard’ sliding directions [25].

**Figure 2:**
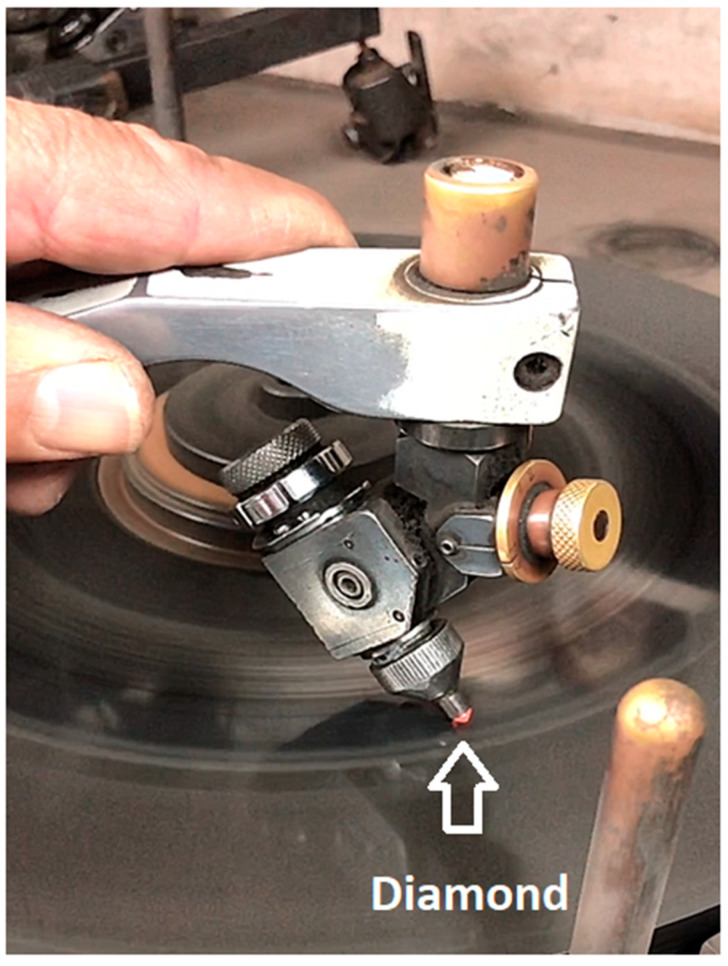
The polishing process of a rough diamond.

The polished layer’s structure differs from that of the underlying bulk material [26]. The bulk temperatures of diamonds undergoing polishing in ‘soft’ directions can typically reach 550–600 K, whereas hard directions might induce higher temperatures. However, the heated diamonds’ visible orange light emission, shown in Figure 2, suggests that the hot spot temperatures during the polishing process are at least ~1150 K [27] (the temperatures generated during polishing also follow the wear anisotropy that can, in part, be attributed to frictional anisotropy [28]).

Thermal or annealing treatment is a controlled heating and cooling process that is sometimes used post-irradiation to change the diamond’s color. The treatment is finished when the desired result is reached (a change in color, for example). This process may induce relaxation of some paramagnetic centers or create new centers (if enough energy is applied). Thermal treatments are used to improve the color quality of the diamond or to bleach undesired colors. These treatments are carried out at a temperature range of 400–1300 °C in the absence of molecular oxygen (in order to avoid oxidation of the diamond) [29].

This study aimed to investigate the effect of applying high-energy conditions, such as the polishing process and high temperature treatments (650 °C and 1000 °C), on the diamonds, which can affect the concentration and nature of the paramagnetic centers in the lattice.

## 2. Materials and Methods

**Figure 3:**
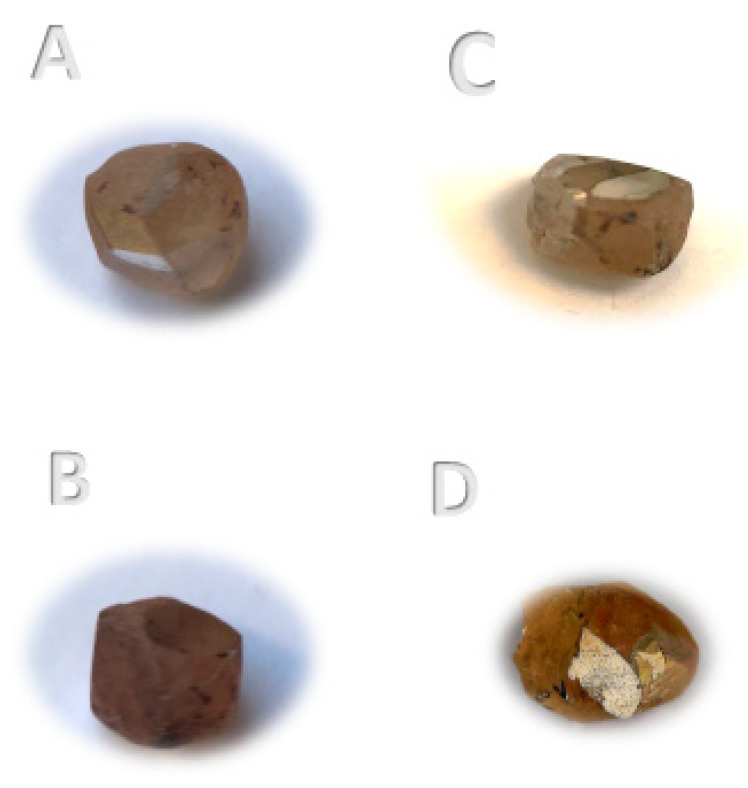
Photographs of the untreated rough diamonds: A–D.

**Diamonds:** Four rough diamonds (A-0.220ct, B-0.220ct, C-0.250ct, and D-0.3015ct) were purchased from the Israeli Diamond Bourse in Ramat Gan for this study (Figure 3).

### 2.1. Spectroscopy

The infrared spectrum of every diamond and the electron paramagnetic (EPR) spectrum were measured, and the fluorescence was observed.

**FTIR** spectra in the range of 500–4000 cm^−1^ were measured using a Alpha2 FTIR spectrophotometer with a resolution of 2 cm^−1^ (Bruker, Karlsruhe Germany), equipped with a ZnSe beam splitter 24 scans were used for each sample. Briefly, the diamond was placed on a gold-coated surface in a DRIFT unit. The bulk nitrogen concentration was calculated by FTIR spectroscopy, taking into account the C–N bond absorption in the 1000–1400 cm^−1^ spectral region [15]. The nitrogen concentration was calculated using the QUIDDIT program reported by Spiech 2020 [30].

**CW-EPR** (continuous wave EPR) spectra were recorded using an E500 Elexsys spectrometer operating at 9.0–9.5 GHz (Bruker, Karlsruhe, Germany). The spectra were recorded at room temperature using a modulation amplitude of 0.3 G, a time constant of 180 ms, and a microwave power range of 20.0 µW. The samples were measured in 5 mm (outside diameter—OD) capillary quartz tubes (Wilmad-labglass, Vineland, NJ, USA). The sample was measured while the diamond‘s sharp end was pointed up. The paramagnetic centers’ concentration was calibrated using Copper (II) sulfate pentahydrate (CuSO_4_) crystal (m = 0.58 mg, Mw = 249.7 g/mole) standards by Strem Chemicals, Inc. The standard spectra were recorded at room temperature using a modulation amplitude of 1 G, a time constant of 80 ms, and an accumulation of 20.0 mW with the same quartz tube. The number of spins was evaluated using a calculation presented in Litvak et al.’s previous study [20].

**Fluorescence** photographs were taken pre- and post-treatment (polish/thermal annealing). The reactions to ultraviolet (UV) light were checked with a conventional four-watt combination longwave (365 nm) device (manufactured by Systems Eickhorst, Hamburg, Germany). Each sample (diamond) was individually projected, and a photograph was taken.

### 2.2. Treatments

**Polishing**: The diamonds were polished to a spherical shape at Meyer Tire Diamond Polishing Ltd. in Netanya, Israel, for about 30 min each, using a grinding machine coated with diamond powder. The diamonds were held via a diamond dop tool with the diameter fitted to the rough diamond. Polishing was conducted on two of the diamonds (A and B). It is important to stress that the orange color of the diamond during the polishing, which indicates high temperature hot spot formation, exists only for a few seconds and immediately when the stone is not pressed, the orange color disappears. This means that during polishing, a high temperature state occurs in the diamond only for short periods.

**Thermal treatment**: The treatment was carried out using a tube furnace with a temperature control system by M.R.C. and was performed on two of the diamonds (C and D) at temperatures of 650 °C and 1000 °C. The quartz glass tube (see Figure 7) containing the diamonds was under vacuum while being heated in order to prevent its reaction with the oxygen in the air, which may result in chemical damage to the diamonds.

## 3. Results and Discussion

**Figure 4:**
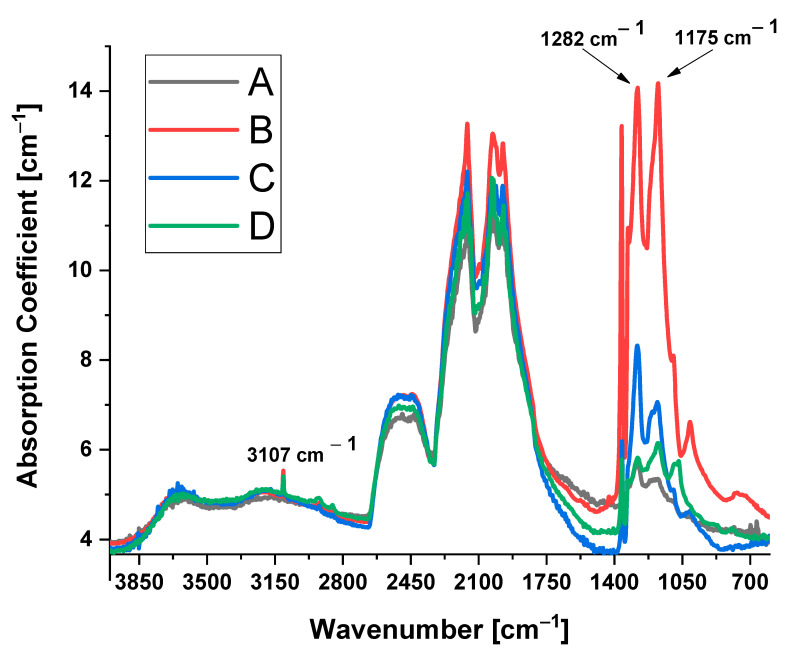
FTIR spectra of the 4 untreated diamonds: A–D.

The FTIR spectra of the four examined diamonds, namely, A, B, C, and D (Figure 4), indicate that all the diamonds contain nitrogen contamination in the range of 39–400 ppm. FTIR spectra and nitrogen concentration were not changed in the rough and polished/thermal treated state; thus, one spectrum for each diamond is presented. The nitrogen contamination consists of type IaA and IaB (absorption peaks at 1282 and 1175 cm^−1^, respectively) aggregates (see Table 1). In all the diamonds an absorption peak at 3107 cm^−1^ was found, which is attributed to H-related vibration and is assigned to the mode of VN_3_H [31].

Polishing was conducted on two of the diamonds (A and B), which resulted in weight reduction of the stones from 0.220 carat to 0.177 carat for A (19.5% weight loss) and 0.156 carat for B (29.1% weight loss).

**Figure 5:**
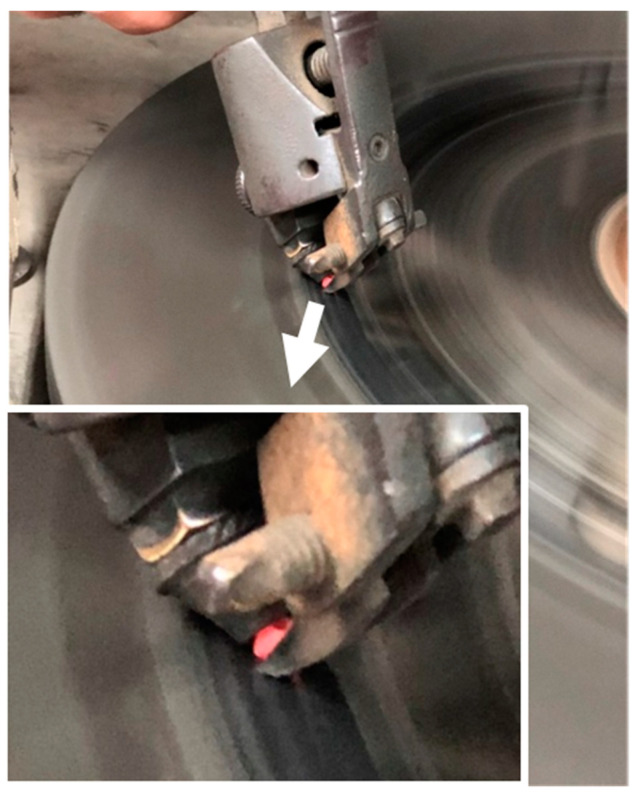
The polishing process of the rough diamond A (insert: the magnification of the “light emission by the heated diamond”).

**Figure 6:**
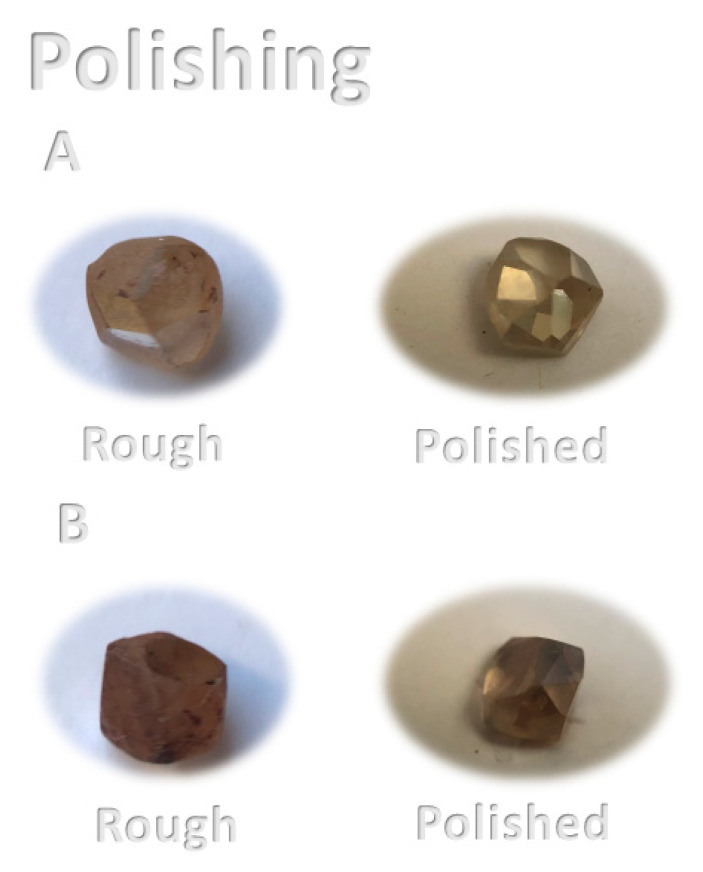
Daylight photographs of the rough A and B diamonds and in their polished state.

Diamond A was photographed during the polishing process (Figure 5); the photograph shows that the diamond emits orange light while being polished because of the high temperature that occurs during the process. Photographs of the pre-treatment (rough) A and B diamonds and the post-treatment (polished) A and B stones are presented in Figure 6. The shape of the polished diamond is spherical and the stones are transparent.

**Figure 7:**
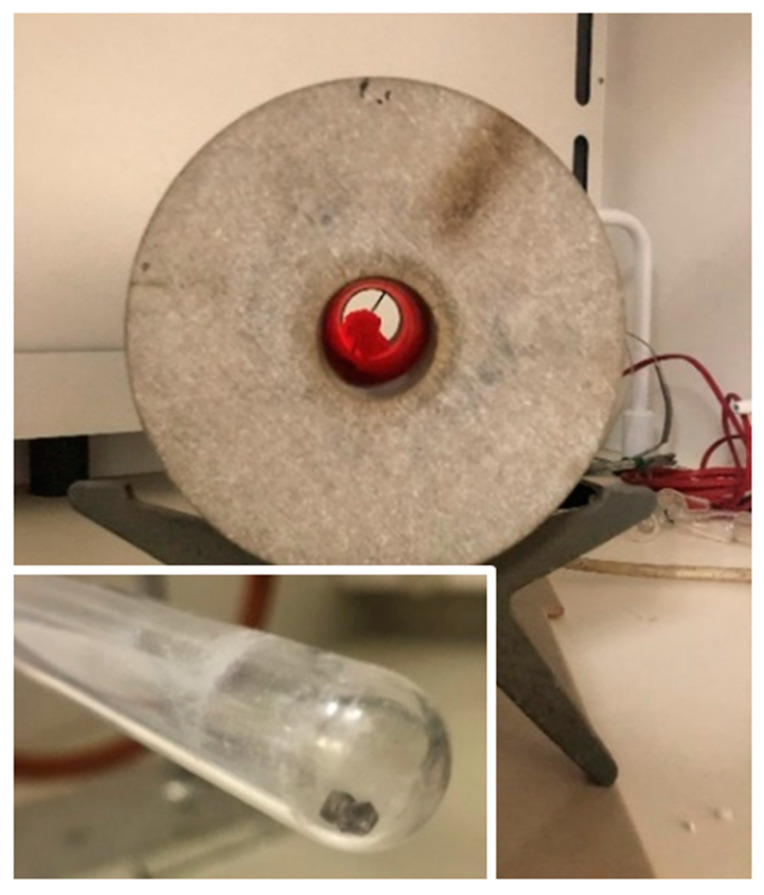
The thermal treatment, the tube furnace glowing at 650 °C, and the magnification of the diamonds post-treatment in a quartz glass tube.

Thermal treatment was carried out on diamonds C and D in a sealed evacuated quartz tube at 650 °C for 20 min; later, the same diamonds were also heated to 1000 °C for 20 min (Figure 7). The glass tube (see the insert in Figure 7) is kept under vacuum while being heated, in order to prevent oxidation with atmospheric oxygen.

**Figure 8:**
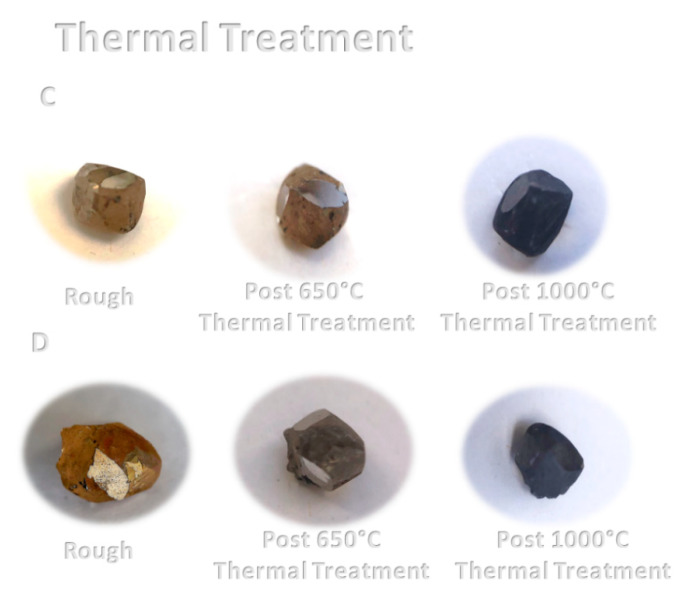
Daylight photographs of the pre- and post-thermal treatment of diamonds C and D.

Photographs of the untreated rough diamonds C and D and following the 650 and 1000 °C thermal treatment are presented in Figure 8; it can be seen that, as the temperature is increased, a dark layer is formed on the surface of the diamonds, as a result of the initial graphitization.

**Figure 9:**
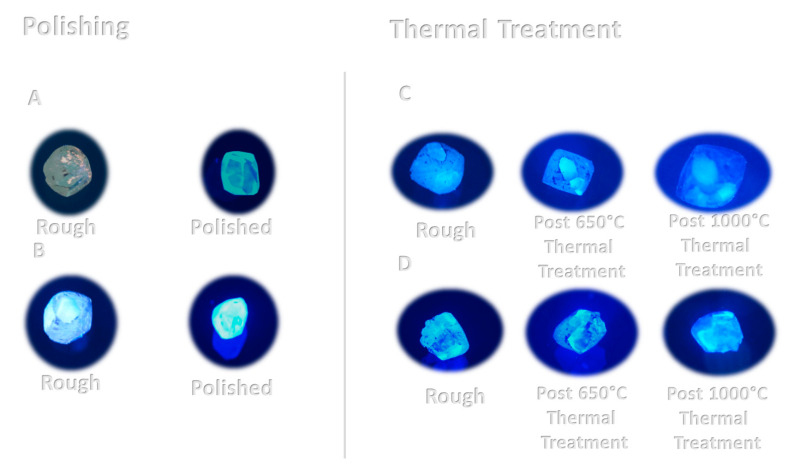
Photographs of the rough and polished (A and B) diamonds and the rough (C and D) non-treated and thermally treated ones.

The 365 nm light excitation fluorescence (Figure 9) did not affect the A diamond; the B diamond in the rough state was characterized by a very light blue fluorescence; however, after the polishing process, a strong blue fluorescence was observed. This observation could result from the following:

(i) The polishing process, which removed the outer shell of the rough diamond, which absorbs the fluorescence light;
(ii) The polishing process created new fluorescence centers.

For the rough diamonds C and D, which had blue fluorescence, no change in the fluorescence pattern was found after the thermal treatments. The blue fluorescence patterns observed here are consistent with the patterns of the Ia-type diamonds described in the literature [15].

**Figure 10:**
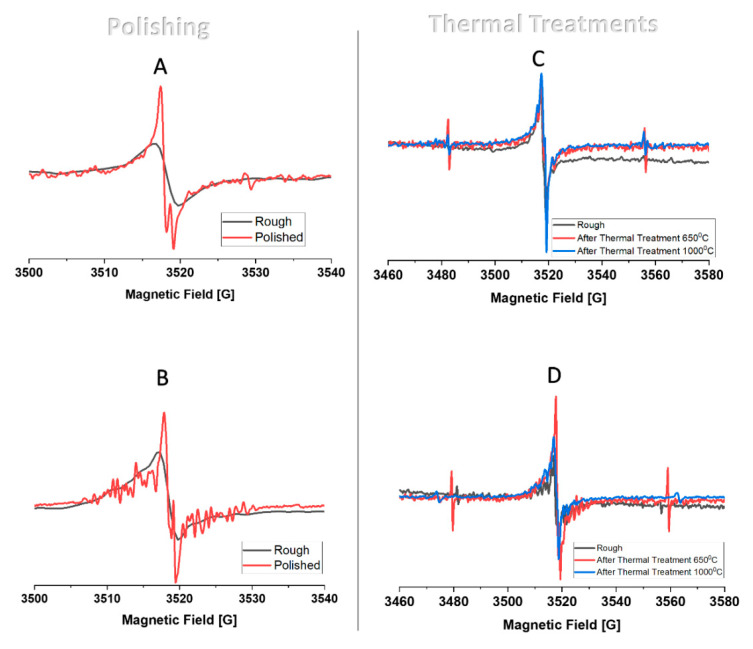
EPR spectra: The effect of polishing (diamonds A and B) and the effect of thermal treatment after 650 °C and 1000 °C (diamonds C and D).

The g values, the paramagnetic centers’ concentration data, and the FTIR nitrogen content evaluation are presented in Table 1 (for A and B) and Table 2 (for C and D). In addition, the EPR spectra of the untreated (rough) and treated (polish/thermal treatment) stones (Figure 10) display a line width reduction after polishing of both diamonds A and B. This means that the polishing eliminated the amorphous structure of the diamond, and thus, a better and sharper analysis could be obtained. Although the polishing process is conducted while the stones are exposed to the air (oxygen), no higher g-values were found (this is attributed to the carbon-centered radicals adjacent to the oxygen atoms). This means that oxygen atoms were not incorporated via the chemical reaction of the hot diamond (during the process) with oxygen atoms [32].

In addition, the spin concentration (#spins/mg) of A and B was reduced by ~20%, probably because of the radical annealing that occurred while the diamond was heated by friction during the polishing process. Moreover, the g value changed from 2.0023(A)/2.0021(B) to 2.0026 (Table 1), which is in agreement with the paramagnetic centers formed by grinding diamonds, as described in the literature (g = 2.0025) [33].

Whereas, in diamonds C and D, there was no significant change in the line width between the pre- and post-thermal treatments—the hyperfine interactions increased because of the thermal treatments. The influence on the hyperfine interactions may be associated with a reorganization of atoms in the lattice during the exposure to high temperature. Consequently, the neighboring nitrogen atoms of the carbon-based paramagnetic centers increase the hyperfine effect and change the EPR spectrum. Spin concentrations, which were calculated for C and D, were found to decrease after thermal treatment of 650 °C (3 to 16%, respectively) and increased after the 1000 °C thermal treatment (20 to 30%). At the lower temperature treatment (650 °C), annealing of some radicals occurs (a high temperature effect) and the temperature is not high enough to produce new radicals [34]. However, at a higher temperature (1000 °C) there is sufficient energy to generate new radicals (paramagnetic centers); the net change in the radicals’ concentration (an increase via radical formation and a decrease via radical annealing) results in an increase in the radicals’ concentration.

The thermal treatment impact can also be confirmed by the change in the g-value from 2.0025 to 2.0024 after 650 °C to 2.0021(C)/2.0023(D) after 1000 °C (Table 2).

The overall results reveal two significant findings: first, the effect of temporary (several seconds) high temperature hot spots and friction (the polishing process) and much longer (isothermal, 20 min per treatment) high temperature (thermal treatment) induce the annealing process (a decrease in radical content) or the formation (an increase in radical content) of the paramagnetic centers’ concentration. Second, the polishing process increases the g-value in both diamonds A and B, whereas thermal treatment decreases the g-value in both diamonds C and D—meaning that the additional factor of the polishing (friction) by itself is an important influence that affects the characteristics of the paramagnetic center generated.

## 4. Conclusions

The purpose of this study was to explore how polishing and thermal treatments affect the carbon-centered radicals in rough diamonds. For this purpose, four diamonds were selected and were subjected to two different treatments: two diamonds were polished and two were thermally treated. The results of the study revealed the following:

(a) The polishing process and the thermal treatment at 650 °C reduced the concentration of the stable paramagnetic centers (stable radicals) due to the annealing process. However, the thermal treatment at 1000 °C increased their concentration via the formation of new paramagnetic centers due to the high temperature induced.
(b) The polishing process reduces the spin concentration and the EPR line width probably just on the surface, since the surface paramagnetic centers are heated much faster than the bulk radicals.
(c) The main difference between the temperature profile during polishing and the thermal treatment is that the polishing process produces short lifetime hot spots (several seconds duration), whereas thermal treatment is a long-lasting high temperature process (30 min).
(d) Thermal treatment results in some reorganization of the nitrogen atoms, which increases the hyperfine interactions between the carbon-based paramagnetic centers and the nearby nitrogen atoms.
(e) It is possible that the polishing process does not only change and anneal some of the paramagnetic center’s structure—in addition, it may also create new fluorescence centers. However, this notion should be further tested in order to corroborate it.

## Figures and Tables

**Table 1:** Rough and polished diamonds’ (A and B) properties of weight, nitrogen, spin concentration, and g-value.

	Weight [ct]		Nitrogen Concentration [ppm]			Spin Concentration [#Spins10^16/mg] {std: ±4.6 × 10^15^}		g-Value	
	Rough	Polished	NA	NB	Ntot	Rough	Polished	Rough	Polished
**A**	0.22	0.18	37	2	39	1.31	0.95	2.0023 ± 0.00004	2.0026 ± 0.00001
**B**	0.22	0.16	139	261	400	9.19	7.30	2.0021 ± 0.00008	2.0026 ± 0.00003

**Table 2:** Rough diamonds’ (C and D) properties of nitrogen, spin concentration, and g-value affected by thermal treatment (650 °C and 1000 °C).

	Weight [ct]	Nitrogen Concentration [ppm]			Spin Concentration [#Spins*10^16/mg] {std: ±3.6 × 10^15^}			g-Value		
		NA	NB	Ntot	Rough	Post 650 °C	Post 1000 °C	Rough	Post 650 °C	Post 1000 °C
**C**	0.25	80	91	171	3.16	3.06	4.03	2.0025 ± 0.00003	2.0024 ± 0.00002	2.0021 ± 0.0002
**D**	0.3	3	117	120	4.70	3.94	4.74	2.0025 ± 0.00001	2.0024 ± 0.00005	2.0023 ± 0.0002

{**NA**—nitrogen concentration of type IaA; **NB**—nitrogen concentration of type IaB; **Ntot**—total nitrogen concentration}.
